# Drivers’ Visual Behavior-Guided RRT Motion Planner for Autonomous On-Road Driving

**DOI:** 10.3390/s16010102

**Published:** 2016-01-15

**Authors:** Mingbo Du, Tao Mei, Huawei Liang, Jiajia Chen, Rulin Huang, Pan Zhao

**Affiliations:** 1 Department of Automation, University of Science and Technology of China, Hefei 230026, China; dumingbo@mail.ustc.edu.cn (M.D.); ruling@mail.ustc.edu.cn (R.H.); 2 Institute of Applied Technology, Hefei Institutes of Physical Science, Chinese Academy of Sciences, Hefei 230026, China; tmei@iim.ac.cn (T.M.); hwliang@iim.ac.cn (H.L.); jjchen@hfcas.ac.cn (J.C.)

**Keywords:** motion planning, autonomous vehicle, drivers’ visual behavior, RRT (rapidly-exploring random tree), on-road driving

## Abstract

This paper describes a real-time motion planner based on the drivers’ visual behavior-guided rapidly exploring random tree (RRT) approach, which is applicable to on-road driving of autonomous vehicles. The primary novelty is in the use of the guidance of drivers’ visual search behavior in the framework of RRT motion planner. RRT is an incremental sampling-based method that is widely used to solve the robotic motion planning problems. However, RRT is often unreliable in a number of practical applications such as autonomous vehicles used for on-road driving because of the unnatural trajectory, useless sampling, and slow exploration. To address these problems, we present an interesting RRT algorithm that introduces an effective guided sampling strategy based on the drivers’ visual search behavior on road and a continuous-curvature smooth method based on B-spline. The proposed algorithm is implemented on a real autonomous vehicle and verified against several different traffic scenarios. A large number of the experimental results demonstrate that our algorithm is feasible and efficient for on-road autonomous driving. Furthermore, the comparative test and statistical analyses illustrate that its excellent performance is superior to other previous algorithms.

## 1. Introduction

Autonomous vehicle technologies have a powerful potential in innovating the automobile industry as well as in improving driving safety. Autonomous driving technology is an emerging field of research that attempts to accomplish its aim of driving safely and comfortably. This field has made considerable progress over the past few years [1,2,3,4,5,6,7,8,9,10,11,12]. As the autonomous driving technology progresses, autonomous vehicles have become one of the most important matters supporting people’s daily life, the economy, and military activities.

In particular, the Defense Advanced Research Projects Agency (DARPA) Grand Challenge and Urban Challenge have greatly promoted research interests in this field. Recently, Google unmanned vehicle already received the qualification of self-driving according to Michigan’s local legislation. Although these advanced vehicles demonstrate outstanding autonomous driving performance, significantly challenging problems in terms of technology and cost continue to exist for fully realizing their commercialization [4,5,6].

The development of autonomous vehicles requires state-of-the-art technologies in perception, decision-making, motion planning, and control. Especially, motion planning plays a vital role in the complete system of an autonomous vehicle by solving the problem of searching for a feasible trajectory to reach a goal position specified by the higher decision-making level. And this trajectory must consider the vehicle dynamics, its maneuver capabilities in the presence of obstacles, along with road boundaries and traffic rules [13]. For the motion planning of autonomous on-road driving, existing planning methods originate primarily from the field of mobile robotics [13], and they have been subsequently applied to different on-rod and off-road vehicles and operational environments [14,15,16,17]. Over the past decade, numerous motion planning algorithms (e.g., potential-field methods, grid-based methods, sampling-based methods) have been proposed in the robotics literature [1,2,3,4,5,6,10,11,12]. Potential-field algorithms assume repulsive forces to obstacles and attractive forces to the goal position [11]. The gradient of the potential field is structured according to the forces. A path can be planned along the steepest gradient of this potential field. However, this path can be easily trapped into the local minima of the potential field [4]. In the grid-based methods, the surrounding environment of the robot is mapped to a set of grid cells, where each grid cell represents an existing obstacle at that grid position in the environment. The optimal path search methods, such as the A* and Anytime dynamic A* algorithms, are usually used to determine a globally optimal path that connects each grid cell from an initial position to a goal position while avoiding static and dynamic obstacles in the environment [12,18]. However, the quality of grid-based algorithm is largely restricted to the grid resolution. In addition, since the memory usage grows exponentially with respect to the complexity of the problem or dimensionality of the state-space, so does the running time of the algorithm [8].

Although these traditional methods are able to find feasible trajectories, the complicated dynamic and differential constraints of the vehicular system are difficult to satisfy. Recently, incremental search methods, such as Probabilistic Roadmap (PRM) or Rapidly-exploring Random Trees (RRT), have received a considerable amount of attention because they have been designed and successfully applied to motion planning in a variety of environments [13]. In particular, RRT [9] is one of the most widely-used algorithms to the robotic motion planning problem. The major advantage of RRT is its applicability to general dynamical system [2], which easily considers the environmental constraints. However, the practical application of this sampling-based method to robotic vehicles is far from straightforward. Therefore, several variants of RRT were proposed for improving it. Kuffner and LaValle presented a bidirectional search with two trees (Bi-RRT) to improve the efficiency [19], but handling the discontinuity of the two search trees proved to be difficult. Environment-guided RRT (EG-RRT) algorithm has been presented and applied in real-world scenes [20]. Particle RRT (pRRT) introduced the particle filter into the RRT algorithm [21], which can sort out the most promising nodes from the distribution set of state rather than a single node. The pRRT algorithm is applied to a Mars exploration rover instead of a real-time autonomous vehicle because it is time-consuming. In 2009, Kuwata introduced a closed-loop prediction model into the framework of RRT (CL-RRT), and this incremental real-time motion planning algorithm has been used in MIT’s autonomous vehicle [2]. Recently, Karaman proposed an asymptotically optimal RRT algorithm (RRT*) that can generate an asymptotic optimal trajectory [22], and RRT* is applied successfully in off-road vehicles [23]. Nevertheless, the characteristic of asymptotic optimal requires more additional time to rewire the tree. Subsequently, Ma *et al.* presented a fast RRT-based variant algorithm, and a rule-template set based on the traffic scenes is used to improve the performance of the algorithm [24].

The aforementioned RRT-based methods, however, continue to exhibit certain deficiencies in terms of motion planning of autonomous vehicle.

- RRT-based planners always generate jerky and unnatural trajectories that contain a number of unnecessary turns or unreachable positions [8]. These issues negatively affect the operations of autonomous vehicles because of the limited turning capability of the vehicles. For example, making a simple lane keeping maneuver on road, it is difficult to plan a straight lined trajectory for RRT-based planners. Especially, the problems become increasingly serious when driving on curved roads, which sometimes even result in the vehicle leaving the roadway.
- The key point of motion planning for autonomous vehicle is real time, whereas the existing RRT-based algorithms consume considerable time to draw useless samples, which decline the overall efficiency. For this issue, the approach in [2] employs different bias sampling strategies according to the various traffic scenes. However, this approach cannot cover every traffic scene, and the related parameters of the sampling lack universality.
- The trajectories planned by RRT barely consider the curvature continuity. For the practical application, this issue can result in the control system problems of autonomous vehicles such as instability, mechanical failure, and riding discomfort. Furthermore, this issue can negatively affect the trajectory tracking of low-level controls, thus increasing the tracking error and controller effort.

**Figure 1:**
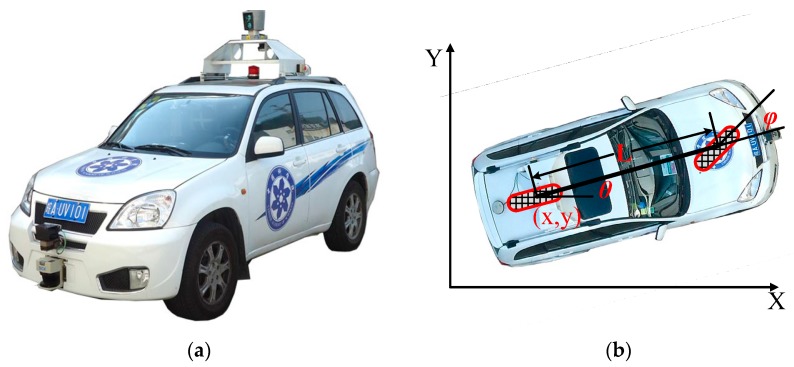
Autonomous vehicle prototype and its kinematic model. (**a**) “Intelligent Pioneer II” and (**b**) autonomous vehicle’s bicycle model.

The motion planner of an autonomous vehicle should ensure safety and comfort of the passengers. Furthermore, the motion planner should also put the vehicle in the right behavior with respect to the kinematic and dynamic model constraints [13]. To overcome the aforementioned issues, this paper focuses on developing a Drivers’ Visual Behavior-guided RRT motion planner (which is abbreviated as DV-RRT in the following sections) to solve the motion planning problem of autonomous on-road driving. The primary novelty of this planner is in the use of the drivers’ visual behavior in the framework of RRT. In the last few decades, numerous behavioral scientists were devoted to studying the link between the driver’s vision positioning and steering behavior when driving down a curved road [25,26,27,28,29,30,31,32]. As a result, they discovered that the drivers use both a “near point” and a “far point” to safely drive through curved roads [26,29]. Therefore, this paper utilizes this characteristic of drivers’ visual search behavior shown on roads to quickly and effectively guide the RRT motion planner. Furthermore, a post-processing method based on B-spline is used to generate smooth, continuous, and executable trajectories in the final stage of the motion planner. To verify our method, the proposed motion planner is implemented and tested on a real autonomous vehicle “Intelligent Pioneer II” (see Figure 1a). A large number of experimental results illustrate that this motion planner can efficiently plan a smooth and reasonable trajectory on roads; meanwhile, the planned trajectory meets the requirement of riding comfort to a certain extent.

The rest of the paper is organized as follows. Section 2 provides a brief survey of preliminary work, including the problem formulation of motion planning according to the autonomous vehicle, Drivers’ Visual Search Behavior on roads, and the calibration between camera and (three-dimensional) laser radar sensor. After reviewing the basic RRT algorithm, Section 3 introduces the DV-RRT algorithm, and then gives a description of effective hybrid sampling strategies and post-processing method in detail. Section 4 reports the experimental results in a real autonomous vehicle and comparison with reference algorithms. Section 5 concludes.

## 2. Preliminary Work and Problem Formulation

In this section, the motion planning problem is formulated at first and then the drivers’ visual search behavior is discussed. Finally, the calibration method between camera and (three-dimensional) laser radar is simply described.

### 2.1. Motion Planning Problem Formulation

For clarity, we first give some definitions for the motion planning problem. The state space and the control space of the vehicle system are represented by compact sets $C\subset {\mathbb{R}}^{n}$ and $U\subset {\mathbb{R}}^{m}$ , respectively. The vehicles have nonlinear dynamics

(1) $$\dot{q}\left(t\right)=f\left(q\left(t\right),u\left(t\right)\right),\;q\left(0\right)=q_{0}$$

where q(t)∈C and u(t)∈U are the states and inputs of the vehicular system, and $q_{0}\in C$ is the initial state at t=0 . Then, the goal state is represented as $q_{goal}\in C$ . The environmental constraints (e.g., static and dynamic obstacles) and vehicle kinodynamic constraints are imposed on the states and inputs, hence, we define the free state space $C_{free}$ to be the set of all free states in C . That is:

(2) $$q\left(t\right)\in C_{free},\;u\left(t\right)\in U$$

Therefore, the obstacle space $C_{obs}$ is defined to be the complement of C , namely, $C_{obs}=C∕C_{free}$ . Thus, the motion planning problem is to determine a trajectory connecting $q_{0}$ with $q_{goal}$ , that is:

(3) $$\{\begin{array}{c} q\left(t\right)\in C_{free},\;\forall t\in \left[0,t_{f}\right]\\ q\left(t_{f}\right)=q_{goal}\;\\ u\left(t\right)\in U,\;\forall t\in \left[0,t_{f}\right] \end{array}$$

where q(t) is referred to as the trajectory and u(t) is referred to as the feasible control input sequence. When t reaches the final time $t_{f}$ , the robotic system can reach the desired goal state $q_{goal}$ .

### 2.2. Vehicle Kinematic Model

The autonomous vehicle prototype “Intelligent Pioneer II”, as shown in Figure 1a, is developed for the Intelligent Vehicle Future Challenge Competition in China. Based on the assumption that the wheels are rolling without slipping and the steering angle is simplified as a single wheel in the midpoint of the two frond wheels [33], the vehicle kinematic model can be described as the bicycle model (see Figure 1b). The kinematic model of the driving vehicle is written as:

(4) $$\left[\begin{array}{c} \dot{x}\\ \dot{y}\\ \dot{\theta }\\ \dot{\varphi } \end{array}\right]=\left[\begin{array}{c} \cos\theta \\ \sin\theta \\ \frac{\tan\theta }{L}\\ 0 \end{array}\right]v+\left[\begin{array}{c} 0\\ 0\\ 0\\ 1 \end{array}\right]\dot{\varphi }$$

where (*x*, *y*) is the Cartesian coordinates of the middle point of the rear wheel axis, *θ* is the angle of the vehicle body with respect to the **X**-axis, *ϕ* is the steering angle, and *L* is the vehicle wheel base. Moreover, *v* and $\dot{\varphi }$ are the longitudinal velocity and the angular steering velocity, respectively. For the experimental testing, the following related boundary parameters of the vehicle are obtained.

(5) $$\begin{array}{c} \text{L}=2.510(\text{m})\\ v_{\min}=0(\text{m}/\text{s}),v_{\max}=12(\text{m}/\text{s})\\ \varphi_{\min}=-0.5236(\text{rad}),\varphi_{\max}=0.5236(\text{rad})\\ {\dot{\varphi }}_{\min}=-0.2183(\text{rad}/\text{s}),{\dot{\varphi }}_{\max}=0.2183(\text{rad}/\text{s}) \end{array}$$

### 2.3. Drivers’ Visual Search Behavior

**Figure 2:**
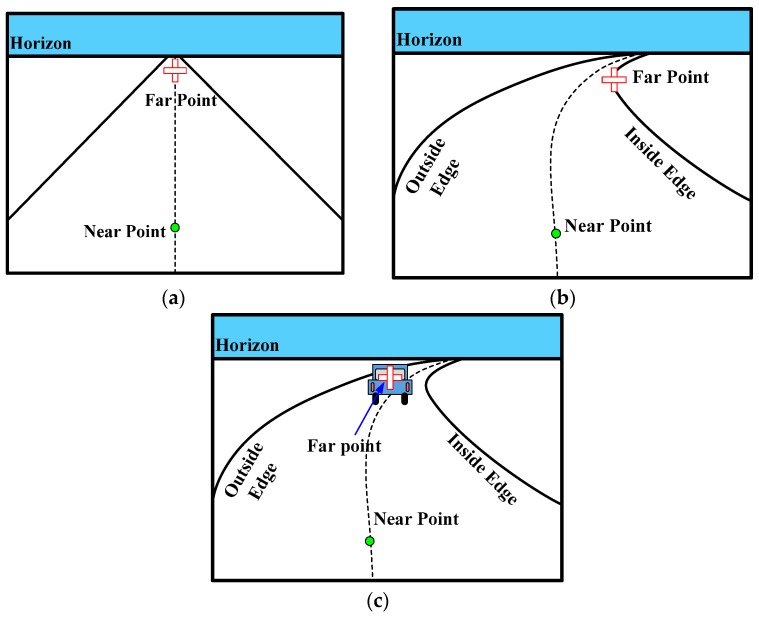
Near and far point for three common scenarios: (**a**) straight road with vanishing point, (**b**) curved road with tangent point; and (**c**) presence of a lead car.

Where a driver looks when he drives is an interesting question. For the past 20 years, the on-road studies of visual behavior in curve driving have become increasingly attractive [28]. Experts and scholars have made a considerable number of important achievements in this field [25,26,27,28,29,30,31,32,33,34]. Most studies have concluded that a driver uses both a near point in the region and a far point in the far region of the roadway to successfully driving through the curved roads. The near and far points are illustrated in Figure 2. The near point (shown with green circles in Figure 2) represents the center of the road at a short distance ahead of the vehicle. The far point (shown by the red cross in Figure 2) is any salient point in the visual scene when driving on roads, such as the vanishing point (shown by the red cross in Figure 2a), tangent point (shown by the red cross in Figure 2b) or, if present, a lead car (depicted by the red cross in Figure 2c). The near point can retain both lateral position and stability of the vehicle, and the far point can maintain a predictive driving heading that compensates for the upcoming road profile [28]. These studies confirm that using these salient points (*i.e.*, near and far points) while driving down the roads is an effective strategy for drivers.

Inspired by the studies of drivers’ visual behavior, the aforementioned near and far points can be introduced into the framework of RRT planner. This paper uses these special points to effectively guide the sampling of RRT. At the same time, the strategy of search is reasonable and human-like, thus making the planned trajectory smooth, which meets the requirements of real-time or riding comfort.

### 2.4. Calibration between Camera and Laser Radar

The solution of the RRT motion planning problem is based on the perception environment map, which is built by cameras and laser radar sensor [35]. To apply the drivers’ visual behavior to the RRT motion planning, the calibration between camera and laser radar is necessary.

**Figure 3:**
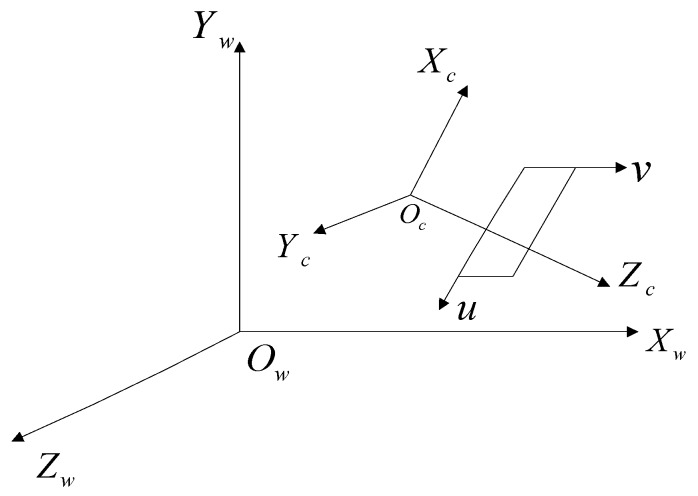
Illustration of between the world coordinate and the camera coordinate.

**Figure 4:**
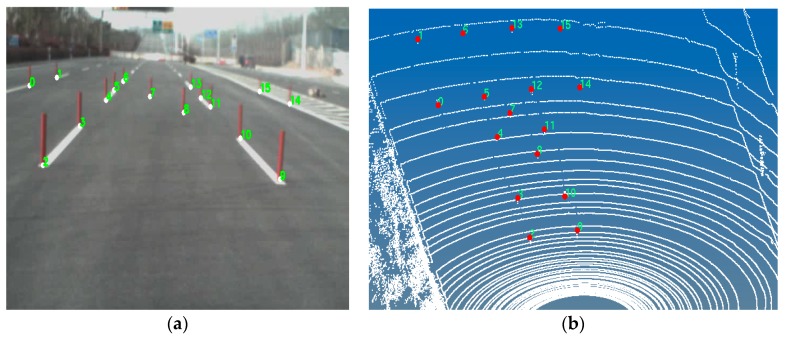
Mapping illustration of between the image data and the laser radar data. (**a**) Image points in the camera coordinate and (**b**) corresponding position points in the world coordinate.

The camera image data and the laser radar data are in different coordinate spaces (shown with Figure 3). Hence, the relationship between the image coordinate and the world coordinate should be derived, the image points in the camera coordinate can be transformed to those in the world coordinate (illustrated by Figure 4). This relationship can be described as:

(6) $$\left[\begin{array}{cccc} X_{1} & X_{2} & ... & X_{N}\\ Y_{1} & Y_{2} & ... & Y_{N}\\ Z_{1} & Z_{2} & ... & Z_{N}\\ 1 & 1 & ... & 1 \end{array}\right]=\left[\begin{array}{ccc} \begin{array}{c} m_{11}\\ m_{21}\\ m_{31}\\ m_{41} \end{array} & \begin{array}{c} m_{12}\\ m_{22}\\ m_{32}\\ m_{42} \end{array} & \begin{array}{c} m_{13}\\ m_{23}\\ m_{33}\\ m_{43} \end{array} \end{array}\right]\left[\begin{array}{cccc} \begin{array}{c} u_{1}\\ v_{1}\\ 1 \end{array} & \begin{array}{c} u_{2}\\ v_{2}\\ 1 \end{array} & \begin{array}{c} ...\\ ...\\ ... \end{array} & \begin{array}{c} u_{N}\\ v_{N}\\ 1 \end{array} \end{array}\right]$$

where the first term of Equation (6) is the object position data in the world coordinate, the third term of Equation (6) is the object image position data in the camera coordinate, and the second term is the mapping matrix between them. The calibration aims to obtain this mapping matrix and then determine the positions of “near” and “far” points in the world coordinate.

## 3. DV-RRT Algorithm

Here, we first provide a review on the operation of the basic RRT algorithm, present the DV-RRT algorithm, and then describe the algorithm flow in detail.

### 3.1. Basic RRT Operation

The primary operation process of the basic RRT is relatively simple. Given an initial state, $q_{init}\in C$ , and a goal state, $q_{goal}\in C$ , the algorithm incrementally searches state space Χ from initial state $q_{init}$ by iteratively selecting a random node $q_{rand}$ , and steering $q_{nearest}$ toward $q_{rand}$ to produce $q_{new}$ , which is added to the tree. This process continues until either the goal state $q_{goal}$ is reached or the number of max iterations goes beyond. And the random sample $q_{rand}$ is drawn from Χ through a uniform sampling strategy (a purely random manner). Moreover, the metric for the nearest-neighbor selection is the Euclidean distance between sampling points.

### 3.2. Overview of the DV-RRT Algorithm

**Figure 5:**
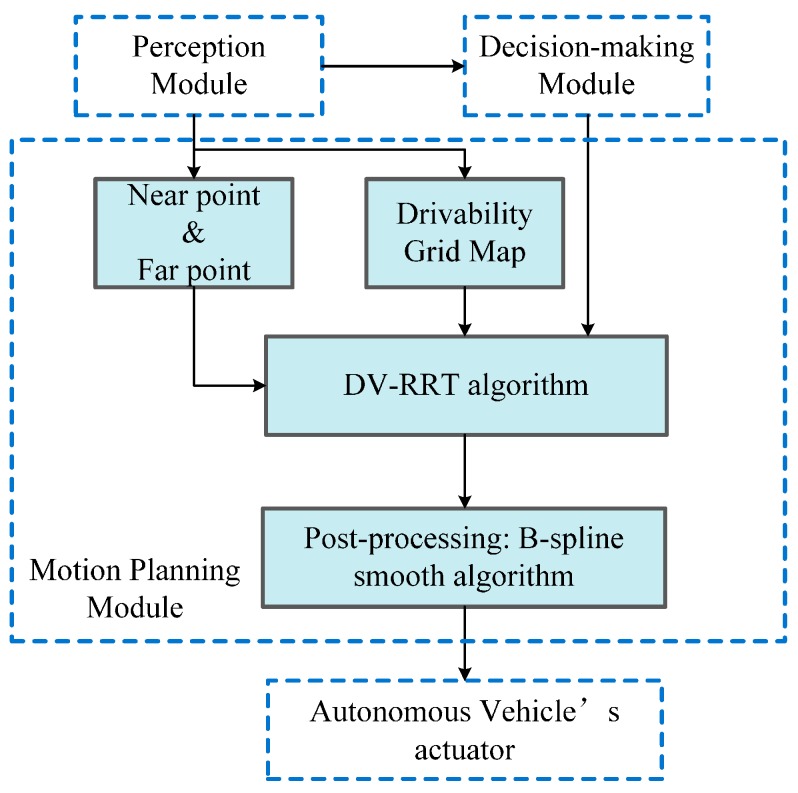
Complete structure of DV-RRT motion planner.

The complete structure of our approach is illustrated in Figure 5. Our approach is developed for the motion planning module of autonomous on-road driving, which receives the environmental information (*i.e.*, Drivability Grid Map, which is described in detail in Section 4.1) and the needed salient points (“near” and “far” points) data from the perception module and the driving maneuver from the decision-making module. The output of our approach is a smooth and executable trajectory, which is converted to a feasible control sequence. Then, the sequence is sent to the vehicle’s actuator. The core of this approach is a RRT variant named DV-RRT algorithm. Based on our preliminary work, this algorithm introduces effective hybrid sampling strategies (detailed in Section 3.3) according to drivers’ visual search behavior. Moreover, a reasonable metric function of nodes connection (detailed in Section 3.4) and a B-spline based post-processing method (detailed in Section 3.5) are employed for further smoothen the trajectory, thus guaranteeing the feasibility of the trajectory.

An overview of the presented algorithm is given in Algorithm 1. In the beginning, the short-term goal states are obtained according to the local road environment (*line 1*). The two salient goal states then guide the growth of two trees (*lines 2–13*). In the process of the growth, a simple and efficient sampling strategy (*i.e.*, using the “near”/“far” point as a bias) is adopted to avoid unnecessary exploration (*line 4*). Then, DV-RRT attempts to connect the sample to the closest node in the tree. To generate a more practical and direct search tree, a reasonable metric function, which considers the turning angle between two nodes, is employed (*line 5*). The termination condition of the loop implies that the maximum number of iterations is exceeded or the goal state is reached (*line 13*). After connecting the two trees (*line 14*), we can obtain a feasible continuous trajectory (*line 15*) by calling the function *Post-processing* ().

**Algorithm 1.** DV-RRT AlgorithmGet near point ***q****_NG_* and far point ***q****_FG_* from perception module.*T_a_* .init (***q****_start_*), *T_b_* .init (***q****_NG_*);**Repeat*****q***_rand1_ ← Get Random Sample 1 (*T_a_*); ***q***_rand2_ ← Get Random Sample 2 (*T_b_*).***q***_near1_ ← Effective_Nearest (*T_a_*, ***q***_rand1_); ***q***_near2_ ← Effective_Nearest (*T_b_*, ***q***_rand2_);***q***_new1_ ← Extend (*T_a_*, ***q***_rand1_, ***q***_near1_); ***x***_new2_← Extend (*T_b_*, ***q***_rand2_, ***q***_near2_);**if** Collision Free (***q***_new1_, ***q***_near1_) **then***T_a_* .Add_Node (***q***_new1_); *T_a_* .Add_Edge (***q***_new1_, ***q***_near1_);**end if****if** Collision Free (***q***_new2_, ***q***_near2_) **then***T_b_* .Add_Node (***q***_new2_); *T_b_* .Add_Edge (***q***_new2_, ***q***_near2_);**end if****Until** find a collision-free path from ***q****_start_* to ***q****_NG_*, and from ***q****_NG_* to ***q****_FG_*;*T* ← Connect (*T_a_*, *T_b_*);**Return** *S* ← Post-processing (*T*);
**Fuction** Effective_Nearest (*T*, ***q***_rand_)
*C*_max_ ← −∞**for** all ***q***_i_ in T*C* ← *C* (***q***_i_, ***q***_rand_) = w_1_ · dis (***q***_i_ , ***q***_rand_) + w_2_ · head (***q***_i_, ***q***_rand_);  **if** *C* > *C*_max_     *C*_max_ ← *C*; ***q***_near_ ← ***q***_i_;  **end if****end for****Return *q***_near_

### 3.3. Effective Hybrid Sampling Strategies

In a structured environment such as on-road driving, sampling the space in random manner can result in large numbers of useless samples [2]. This section presents a simple and efficient sampling strategy that use the “near” and “far” points to bias the different Gaussian sampling clouds to enable real-time generation of feasible trajectory.

For clarity, the definitions of the near and far points are provided at first. The near point is described as a point in the center of the near lane in front of the vehicle, set at a distance of 10 m from the vehicle’s center, whereas the far point is described as one of three objects: (a) the distant point of a straight roadway according to the vehicle’s speed (see Equation (7)); (b) the tangent point of a curved road; and (c) a lead vehicle, that is, the vehicle in front of the autonomous vehicle [26].

(7) $$L_{far}(v)=\{\begin{array}{c} 20,v\leq 5m/s\\ 4v,v>5m/s \end{array}$$

where the unit is meter for *L_far_*.

Each sample point ***q***= [*q_x_*, *q_y_*]^T^ is generated with respect to a certain reference position and heading (*x*_0_, *y*_0_, θ_0_) by:

(8) $$\{\begin{array}{c} q_{x}=x_{0}+r\cdot \cos(\theta )\\ q_{y}=y_{0}+r\cdot \sin(\theta ) \end{array}$$

(9) {r=σr|nr|+r0θ=σθ|nθ|+θ0

where *r*_0_ and θ_0_ are offsets according to (*x*_0_,*y*_0_), *n_r_* and *n*_θ_ are random variables, *σ_r_* and *σ*_θ_ are standard deviation with Gaussian distributions.

**Figure 6:**
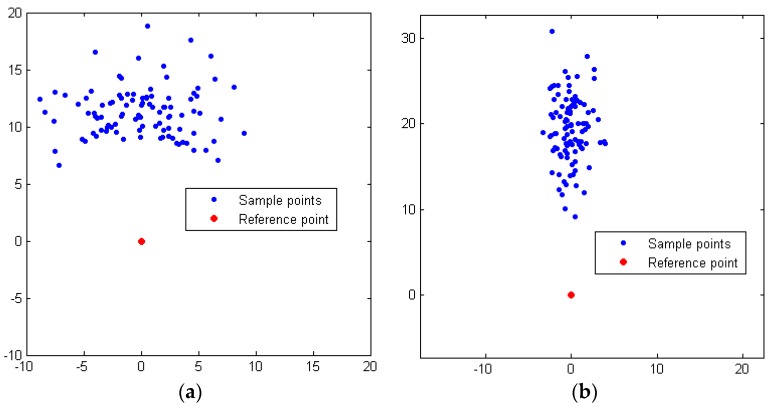
(**a**) Biased Gaussian samplings for near point in a straight road and (**b**) Biased Gaussian samplings for far point in a straight road.

In terms of the near point, a wide and short Gaussian distribution is used to fully exploit the road and save the sampling number because the distance between the vehicle and the near point is relatively short. Note that the vehicle’s current state is set as the reference state at this sampling process, as shown in Figure 6a. In terms of the far point, however, the sampling manner switches to a relatively narrow and long Gaussian distribution because of the road boundary constraints and the driving rules. Additionally, at this time, the near point is set as the reference point of the Gaussian sampling, as shown in Figure 6b.

### 3.4. Reasonable Metric Function

Based on the metric function, the planner connects the random sample to the closest node in the tree. Usually, the Euclidean distance is utilized as metric function to select the closest node, but a smooth extension is needed when RRT is applied to a vehicle with limited turning capability [2]. For vehicle, it favors smoother paths instead of shorter ones. Therefore, the turning angle between two neighboring nodes should be considered (for more detail, the readers can refer to [36]). Thus, a reasonable metric function *C*(*q_i_*, *q_j_*) is presented:

(10) $$\{\begin{array}{l} \;C(q_{i},q_{j})=w_{1}\cdot D(q_{i},q_{j})+w_{2}\cdot H(q_{i},q_{j})\\ \;D(q_{i},q_{j})=N1(\sqrt{{\left(x_{i}-x_{j}\right)}^{2}+{\left(y_{i}-y_{j}\right)}^{2}})\\ H(q_{i},q_{j})=N2(\left|\theta_{i}-\theta_{j}\right|) \end{array}$$

(11) $$\{\begin{array}{c} N_{1}(d)=\frac{d_{\max}-d}{d_{\max}}\\ N_{2}(\theta )=\frac{\theta_{\max}-\theta }{\theta_{\max}} \end{array}$$

where *N*_1_(*d*) and *N*_2_(θ) are the normalized functions of distance and turning angle, respectively. The distance and angle are different dimensions, hence, a simple and effective normalization method is used to handle these two parameters.

### 3.5. B-Spline-Based Post-Processing Method

**Figure 7:**
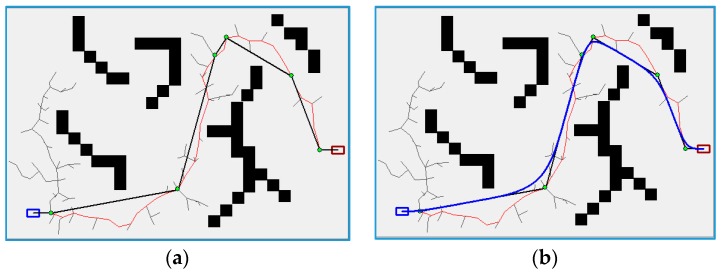
Simulation illustration of post-processing method: (**a**) RRT-based searching path and the corresponding pruning path and (**b**) smoothing path.

It is well known that RRT-based planners often obtain meandering and jerky paths, which are bad for the vehicle to execute [8,19,36]. To solve this problem, a B-spline based post-processing method, which consists of two parts, namely, pruning part and smoothing part, is proposed for generating smooth paths. Algorithm 2 demonstrates the main flow of the post-processing method. Additionally, a simulation illustration of the post-processing method is given, as shown in Figure 7. The red line represents the RRT-based searching path, the black solid line indicates the corresponding pruning path, and the blue solid line illustrates the smoothing path by using B-spline curves.

**Algorithm 2.** Post-Processing Method*Q* ← Pruning (*T*);*S* ← Cubic_Bspline (*Q*);**Return** *S*
**Function** Pruning (*T*)
*T* ← obtained from DV-RRT;Var *Q*_1_, *Q*_2_: Path*Q*_1_ (***q***_0_, ***q***_1_, ***q***_2_, **…**, ***q***_n_) ← Path (*T*);***q***_temp_ ← ***q***_0_; *Q*_2_ Add_Node (***q***_0_);**while *q***_temp_! = ***q***_n_ do**for** each node ***q***_i_ ∈ *Q*_1_**if** Collision (***q***_temp_, ***q***_i_) **then*****q***_temp_ ← ***q***_i_; *Q*_2_ Add_Node (***q***_temp_);**break****end if****end for***Q*_2_ Add_Node (***q***_n_);**end while****Return** *Q*_2_;

The first part of this method is pruning the path by shortcutting it, which is illustrated as a black solid line in Figure 7a. And then, the pruning is needed to convert the linear path to a feasible and continuous path (demonstrated by the blue solid line in Figure 7b). Circular arcs, straight lines and clothoids are often used for continuous path smoothing in robotics [37,38]. However, these methods have shortcomings such as curvature discontinuity, lacking closed form expression, and so on [36]. Therefore, cubic B-spline curves, which can be locally modified without affecting the whole path, are utilized to ensure path continuity. A ***K***-th degree B-spline curve is defined as follows:

(12) $$C(u)=\sum_{i=0}^{n}N_{i,k}(u)\cdot P_{i}$$

where ***N****_i,k_*(*u*) is the B-spline basis function which is defined by Cox-de Boor (Equations (13) and (14)). Control points ***P***_i_ are obtained from the first part of post-processing method (*i.e.*, pruning path).

(13) $$N_{i,0}\left(u\right)=\{\begin{array}{c} 1,u_{i}\leq u\leq u_{i+1}\\ 0,otherwise \end{array}$$

(14) $$N_{i,k}=\frac{u-u_{i}}{u_{i+k}-u_{i}}N_{i,k-1}(u)+\frac{u_{i+k+1}-u}{u_{i+k+1}-u_{i+1}}N_{i+1,k-1}(u)$$

## 4. Experimental Results and Discussion

To verify the performance of the DV-RRT approach, we have implemented it in our autonomous vehicle platform called “Intelligent Pioneer II” (see Figure 1). The vehicle is equipped with a GPS/INS receiver, three LIDAR sensors (two Sick LMS, one Velodyne HDL-64), and three cameras (Imaging Source DFK 22AUC03) [8]. Experiments are conducted under two different types of environments, namely, straight and curved roads with and without traffic vehicles. In curved roads with traffic vehicles, the scenarios with a lead vehicle and static vehicles are set in the test, respectively. During the tests, the vehicle travels at a speed of up to 40 km per hour on straight roads and 30 km per hour on curved roads. The perceptual environment information around the vehicle changes real-time. The experimental results demonstrate the effectiveness of the proposed motion planning method.

### 4.1. Drivability Grid Map

**Figure 8:**
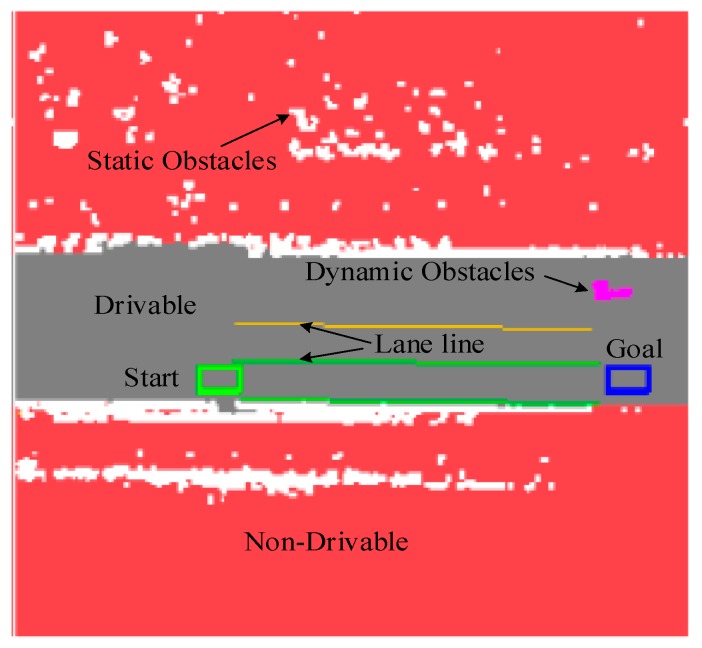
Drivability Grid Map.

To enable the motion planning to interface with the perceived environment [35], the perception data, which includes non-drivable and drivable regions and the static and dynamic obstacles, are rendered into a Drivability Grid Map, as shown in Figure 8. The red part represents the non-drivable region, which are off-limited to the vehicle. The white part indicates the static obstacles, whereas the magenta part indicates the dynamic obstacles. The green rectangle is the current position of vehicle, and the blue rectangle, obtained from the “far” point of drivers’ visual behavior, is the short-term local goal location.

### 4.2. Comparative Test and Analysis

**Figure 9:**
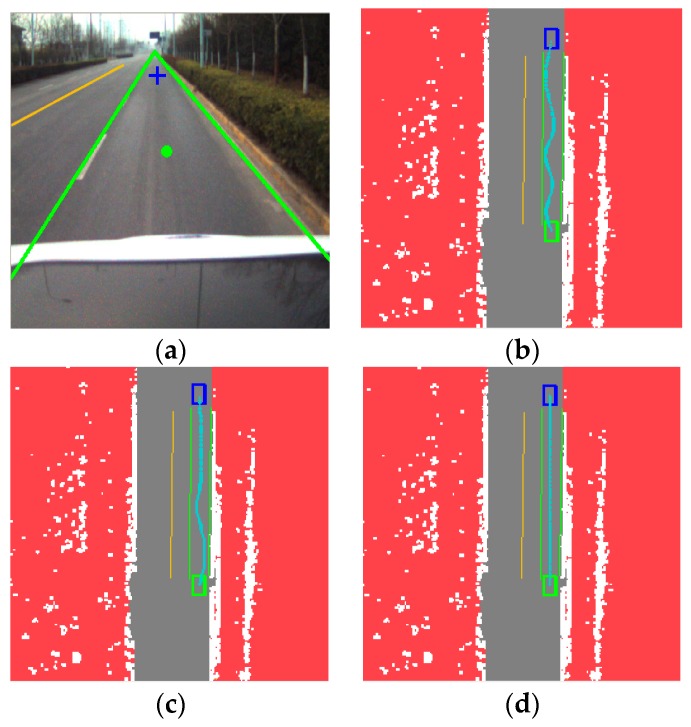
Comparison among different algorithms on a straight road: (**a**) experimental scenario, (**b**) result of basic RRT; (**c**) result of Bi-RRT; and (**d**) result of DV-RRT algorithm.

**Figure 10:**
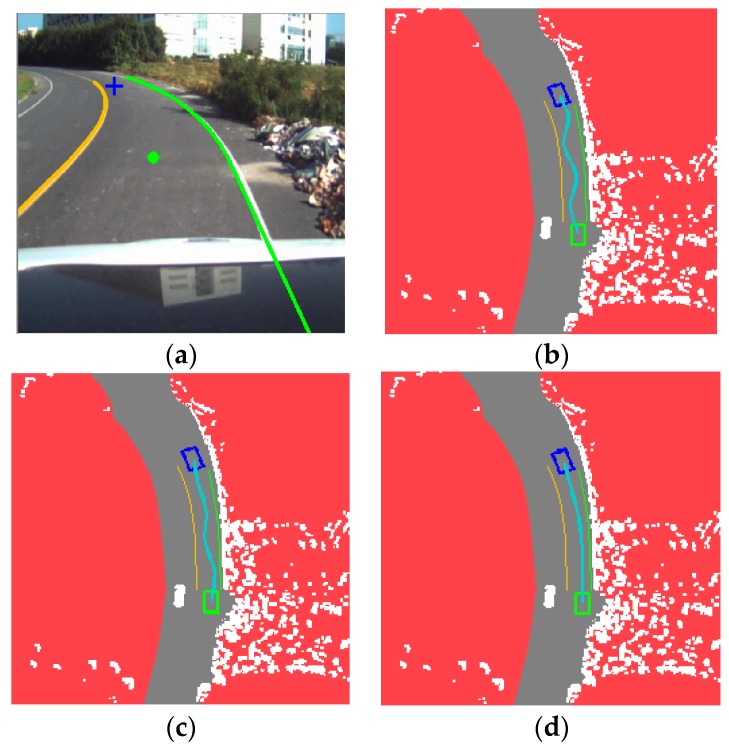
Comparison among different algorithms on a curved road: (**a**) experimental scenario; (**b**) result of basic RRT; (**c**) result of Bi-RRT; and (**d**) result of DV-RRT algorithm.

To clearly demonstrate the advantage of DV-RRT algorithm for autonomous on-road driving, the algorithm is compared with two other related algorithms, namely, basic RRT algorithm and Bi-RRT. Two common scenarios, namely, straight and curved roads are used for test at first. The comparison results of the DV-RRT and related algorithms for straight and curved roads are presented in Figure 9 and Figure 10, respectively.

Driving on a straight road without obstacles is the most common traffic scenario. Figure 9a displays the result of the extracted “near” point (shown with green circle) and “far” point (shown with blue cross) according to the drivers’ visual search behavior on a straight road. Figure 9b presents the planning result of the basic RRT algorithm, which obviously generates a number of unnecessary turns. Figure 9c indicates the result of Bi-RRT. Although the unnecessary turns is relative less, the trajectory is exceedingly close to the road boundary. The result of the proposed approach is illustrated in Figure 9d. On a straight road, DV-RRT can plan a trajectory that is almost a straight line, enabling the intelligent vehicle to travel safely (see Figure 9d). Note that the planning trajectory of each approach is shown with the cyan line in Figure 9.

Figure 10 illustrates that the vehicle negotiates a curved road. Figure 10a visualizes the result of the extracted “near” point (shown with green circle) and “far” point (shown with blue cross) according to the drivers’ visual search behavior on a curved road. Figure 10b indicates the result of the basic RRT under this scenario. The uniform random sampling makes the planned trajectory execute redundant turns. In contrast, Bi-RRT gets a better trajectory due to its bidirectional search, as shown in Figure 10c. However, it still contains less turns and closes to the road boundary. By comparing these results with that of our algorithm (shown in Figure 10d), the result planned by DV-RRT, because of the guide of drivers’ visual search behavior and biased sampling method, exhibits a real trajectory similar to that of a human driver. Moreover, the B-spline based post-processing method makes the trajectory smoother.

**Figure 11:**
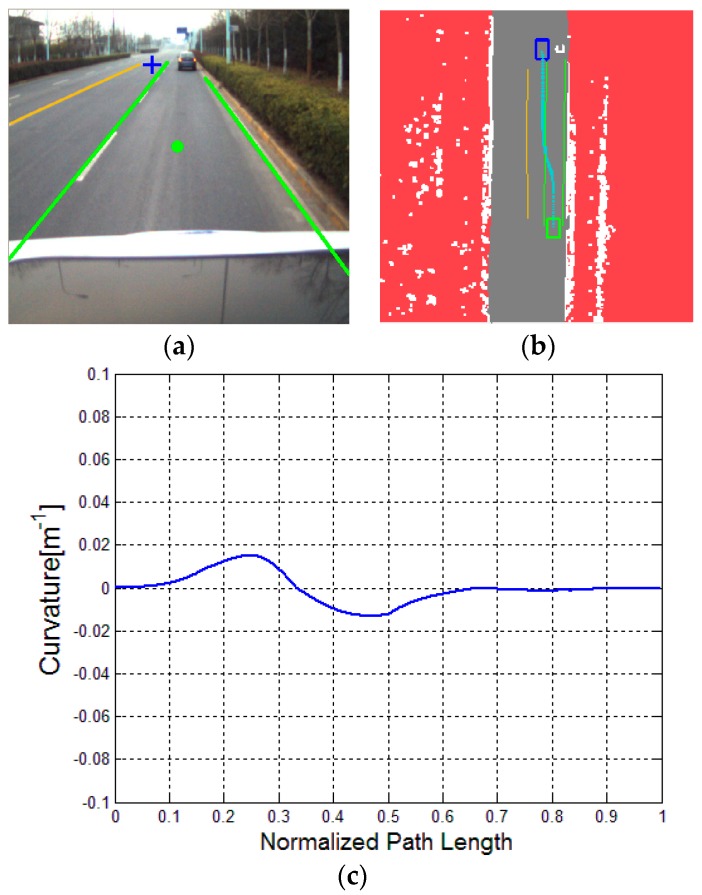
Driving on a straight road with a parked car: (**a**) experimental scenario, (**b**) result of DV-RRT algorithm; and (**c**) corresponding bounded continuous curvature.

**Figure 12:**
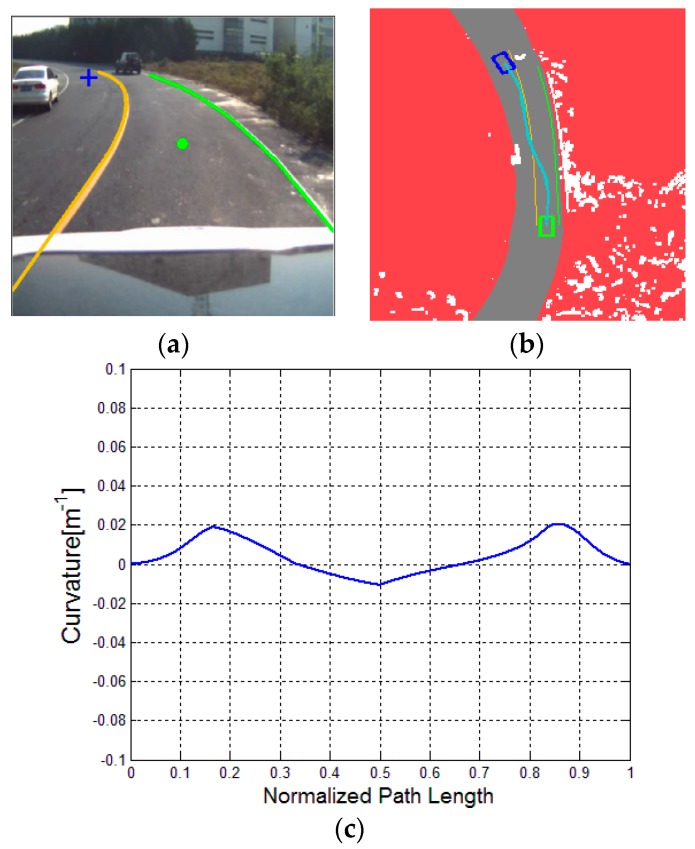
Driving on a curved road with two parked cars: (**a**) experimental scenario; (**b**) result of DV-RRT algorithm, and (**c**) corresponding bounded continuous curvature.

**Figure 13:**
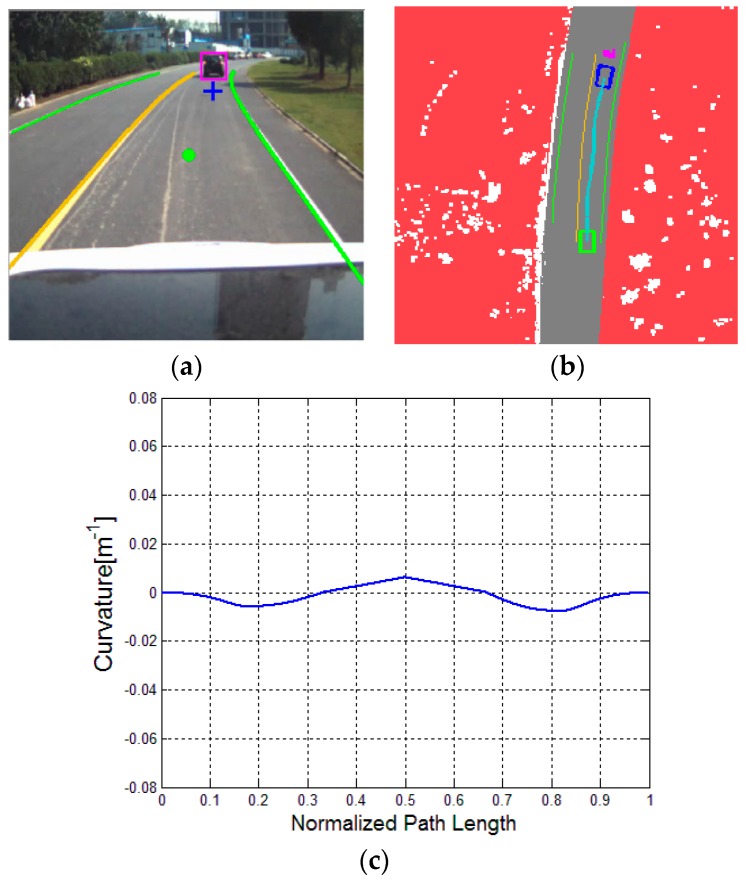
Car following on a curved road with a leader car: (**a**) experimental scenario, (**b**) result of DV-RRT algorithm, and (**c**) corresponding bounded continuous curvature.

To further evaluate the proposed algorithm, the relatively complicated traffic scenarios, namely, a straight road with a parked car, a curved road with parked cars and a curved road with a lead (dynamic) car, are considered. Respectively, the three typical illustrations are presented in Figure 11, Figure 12 and Figure 13.

Figure 11 indicates that the vehicle bypasses a parked car in the current lane. Figure 11a represents the result of the extracted “near” point (shown with green circle) and “far” point (shown with blue cross) according to the drivers’ visual search behavior. The trajectory planned by DV-RRT algorithm easily performs the lane changing maneuver (shown in Figure 11b). Figure 12 illustrates that the vehicle bypasses two parked cars on a curved road using the “near” and “far” points as a guide (shown in Figure 12a). Additionally, the trajectory generated by DV-RRT algorithm is smooth and executable for “Intelligent Pioneer II”(shown in Figure 12b). Figure 13 demonstrates that the vehicle follows a lead car (magenta rectangle shown in Figure 13a or magenta part in Figure 13b) on a curved road by means of the proposed approach. Evidently, the DV-RRT algorithm can accomplish the car-following task easily in this traffic scenario. Moreover, the trajectories planned with DV-RRT algorithm have smooth and continuous curvature (shown in Figure 11c, Figure 12c and Figure 13c) because a reasonable metric function and a B-spline based post-processing method are applied in the frame of the algorithm.

The test results of three different algorithms for the aforementioned five common traffic scenarios are listed in Table 1. To eliminate the randomness of algorithm, each algorithm was tested 500 times. Then, Table 1 provides the relevant indicators (characteristics), including the average number of samples, the average number of nodes, the average running time, the maximum curvature of trajectory and the average path length. Compared with the presented algorithm, the related algorithms (basic RRT and Bi-RRT) require more sampling numbers, cost more runtime and generate more tree nodes, because they get the trajectory through random searching without effective guidance. From the average running time in Table 1, the proposed algorithm exhibits approximately 4.16 times and 2.39 times the improvement over basic RRT and Bi-RRT, respectively. For the five common traffic scenarios, the proposed algorithm can quickly find a smooth and feasible trajectory using the guide of drivers’ visual search behavior. Therefore, the running time of DV-RRT algorithm is very short, which is less than 90 ms. In addition, the maximum curvature of trajectory planned by DV-RRT algorithm is significantly less than those of the basic RRT and Bi-RRT. Furthermore, the length of the trajectory planned by DV-RRT algorithm is obviously shorter than those planned by the two other algorithms in all cases.

**Table 1:** Performance comparison among different methods in various traffic scenarios.

Scenarios	Indicators	Approaches		
		Basic RRT	Bi-RRT	DV-RRT
Scenario 1: Straight road	Average number of samples	351.4	180.5	89.2
	Average number of nodes	163.6	89.7	39.7
	Average running time (ms)	210.5	100.4	43.3
	Maximum curvature (m^−1^)	0.086	0.044	0
	Average path length (m)	43.76	41.81	40.10
Scenario 2: Curved road	Average number of samples	481.7	250.4	110.6
	Average number of nodes	249.5	136.2	50.7
	Average running time (ms)	264.2	160.4	53.1
	Maximum curvature (m^−1^)	0.105	0.066	0.009
	Average path length (m)	33.45	32.61	31.53
Scenario 3: With a parked car on the straight road	Average number of samples	410.3	203.4	130.2
	Average number of nodes	191.6	124.7	64.4
	Average running time (ms)	211.2	143.5	66.7
	Maximum curvature (m^−1^)	0.092	0.078	0.015
	Average path length (m)	43.57	41.63	39.22
Scenario 4: With two parked cars on the curved road	Average number of samples	551.8	326.1	198.5
	Average number of nodes	302.4	172.8	83.6
	Average running time (ms)	330.7	190.2	86.2
	Maximum curvature (m^−1^)	0.112	0.093	0.02
	Average path length (m)	39.01	37.82	36.15
Scenario 5: With a lead (dynamic) car on the curved road	Average number of samples	504.1	297.3	162.4
	Average number of nodes	276.5	158.6	71.9
	Average running time (ms)	290.3	173.7	73.4
	Maximum curvature (m^−1^)	0.097	0.084	0.008
	Average path length (m)	36.58	35.13	33.71

**Figure 14:**
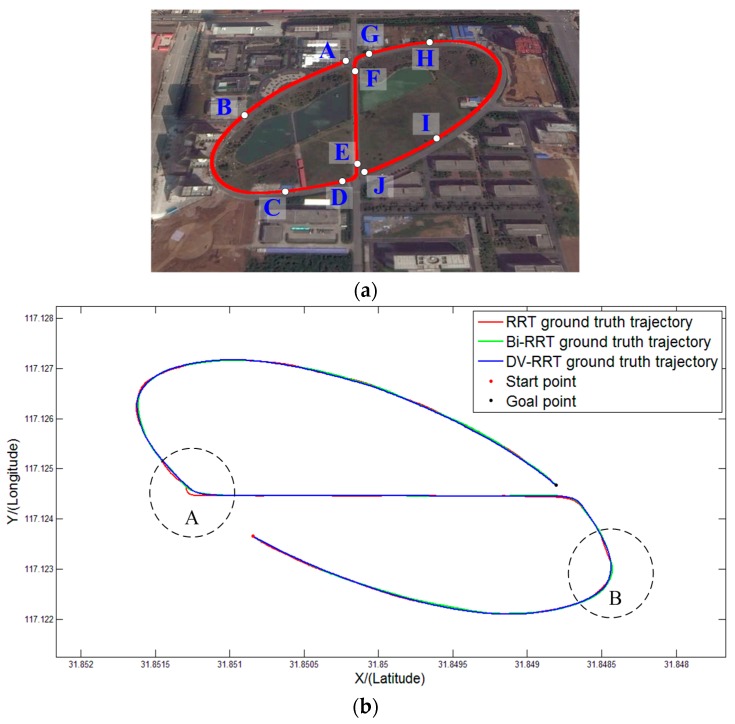

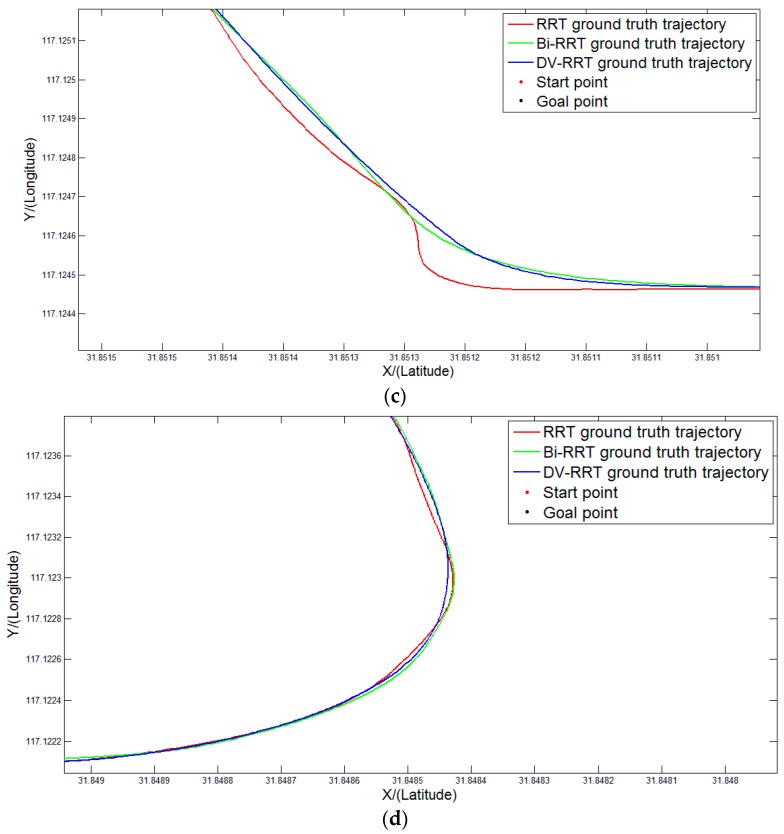
Ground truth trajectories of “Intelligent Pioneer II” recorded during experiment: (**a**) experimental route and way points, (**b**) GPS trajectories of three different algorithms; (**c**) enlarged illustration of **A** area; and (**d**) enlarged illustration of **B** area.

Figure 14 illustrates the ground truth trajectories of “Intelligent Pioneer II” recorded during a comparative test. Although the three different approaches can achieve the test route, their actual navigation performance have obvious differences. Figure 14a represents the well-defined route and the related way points. The comparison result of the three different methods is shown in Figure 14b. Figure 14c,d are the enlarged illustrations of **A** and **B** areas, respectively. The ground truth trajectory of DV-RRT (blue line)is smooth and reasonable (see Figure 14c,d). However, the ground truth trajectories of RRT (red line) and Bi-RRT(green line) always contain numerous unnecessary turns, especially on curved roads (e.g., **A** and **B** areas). The outdoor comparative experiment further validates the proposed approach.

## 5. Conclusions

This paper presents a drivers’ visual behavior-guided RRT (DV-RRT) algorithm to solve the motion planning problem of autonomous on-road driving. The primary novelty of this method is in the use of drivers’ visual search behavior in the framework of RRT planner. This motion planner utilizes the guidance information from the fusion data of the camera and laser radar sensor to acquire efficient sampling and obtain reasonable planned results. The characteristic of the drivers’ visual search behavior is the visual fixation points (“near” and “far” points), which can increase the real-time performance of the algorithm (guiding the sampling process) and mimic human driving on road. To improve the executable capability and smoothness of the trajectory, a reasonable metric function and a B-spline based post-processing method are proposed and used during the planning process.

A large number of tests and statistical analyses were conducted to compare the performance of the proposed algorithm against other related algorithms in different traffic scenarios. Experimental results validate that the presented algorithm can quickly plan a smooth and feasible trajectory. For real-time autonomous navigation on road, the intelligent vehicle can properly react in the presence of static obstacle (parked car) and dynamic obstacle (lead car). Based on the analysis, the introduction of the drivers’ visual search behavior can improve the efficiency of the RRT and ensure the planned trajectory compliance with the road geometric structure and the humans driving behavior.

However, our method has several limitations. In certain structured environments, such as an urban environment, the proposed method can easily accomplish the motion planning with the guidance of clear lane mark and obvious road boundary. With no reliable road boundary and no clear lane mark, however, this method cannot obtain a good result because of the lack of the accurate guidance information. Therefore, for these unstructured environments, such as rural and field roads, we intend to utilize the visual image information from prior vehicle tire tracks and pedestrian footsteps to guide the motion planning. Considering the guidance nature of the drivers’ visual search behavior, it is believed that the proposed algorithm can also be applied to unstructured roads in the near future.

Specifically, in a future work, we intend to utilize the camera as an important sensor to sense the road environment from drivers’ perspective and then develop a cognitive environment model (map) based on the drivers’ visual. Moreover, we intend to research the visual selective attention mechanism in several highly complicated traffic scenarios to further enhance the robustness of our method and apply it to autonomous vehicle for off-road driving. Finally, we intend to further study the multi-sensor information fusion technology, especially between the stereo cameras and laser radar sensors, to improve the sensing capability and intelligence of autonomous vehicles.
